# Bundle-sheath leakiness and intrinsic water use efficiency of a perennial C_4_ grass are increased at high vapour pressure deficit during growth

**DOI:** 10.1093/jxb/erw417

**Published:** 2016-11-18

**Authors:** Xiao Ying Gong, Rudi Schäufele, Hans Schnyder

**Affiliations:** Lehrstuhl für Grünlandlehre, Technische Universität München, Alte Akademie, Freising, Germany

**Keywords:** C_4_ photosynthesis, CO_2_-concentrating mechanism, carbon isotope discrimination, gas exchange, nitrogen nutrition, vapour pressure deficit

## Abstract

Bundle-sheath leakiness (*ϕ*) is a key parameter of the CO_2_-concentrating mechanism of C_4_ photosynthesis and is related to leaf-level intrinsic water use efficiency (WUE_i_). This work studied short-term dynamic responses of *ϕ* to alterations of atmospheric CO_2_ concentration in *Cleistogenes squarrosa*, a perennial grass, grown at high (1.6 kPa) or low (0.6 kPa) vapour pressure deficit (VPD) combined with high or low N supply in controlled environment experiments. *ϕ* was determined by concurrent measurements of photosynthetic gas exchange and on-line carbon isotope discrimination, using a new protocol. Growth at high VPD led to an increase of *ϕ* by 0.13 and a concurrent increase of WUE_i_ by 14%, with similar effects at both N levels. *ϕ* responded dynamically to intercellular CO_2_ concentration (*C*_i_), increasing with *C*_i_. Across treatments, *ϕ* was negatively correlated to the ratio of CO_2_ saturated assimilation rate to carboxylation efficiency (a proxy of the relative activities of Rubisco and phosphoenolpyruvate carboxylase) indicating that the long-term environmental effect on *ϕ* was related to the balance between C_3_ and C_4_ cycles. Our study revealed considerable dynamic and long-term variation in *ϕ* of *C. squarrosa*, suggesting that *ϕ* should be determined when carbon isotope discrimination is used to assess WUE_i_. Also, the data indicate a trade-off between WUE_i_ and energetic efficiency in *C. squarrosa*.

## Teaser

Bundle-sheath leakiness of a perennial C_4_ grass responds dynamically to short-term variation of atmospheric CO_2_ concentration, and is altered by long-term changes of vapour pressure deficit.

## Introduction

The CO_2_ concentrating mechanism (CCM) is a specialized feature of C_4_ photosynthesis and enables the maintenance of a very high CO_2_ partial pressure at the site of ribulose-1,5-bisphosphate carboxylase/oxygenase (Rubisco), thus effectively minimizing photorespiration (Hatch, 1987). The CCM underlies the higher quantum yield, and higher water- and nitrogen-use efficiency of C_4_ plants relative to C_3_ plants under high temperature and/or low intercellular CO_2_ (Ehleringer *et al.*, 1997; Long, 1999). In C_4_ photosynthesis, CO_2_ is first fixed by phosphoenolpyruvate carboxylase (PEPc) in mesophyll cells to form C_4_ acids (the C_4_ cycle), which are transported into bundle-sheath cells, where the acids are decarboxylated and CO_2_ is finally fixed by Rubisco (the C_3_ cycle). Effective CCM requires a strong ‘CO_2_ pump’ (a high rate of the C_4_ cycle). However, leakage of CO_2_ from bundle-sheath cells back to mesophyll cells represents an energetic inefficiency that is related to the cost of the regeneration of phosphoenolpyruvate (PEP; Hatch, 1987; Furbank *et al.*, 1990). The ratio of this leakage rate to the C_4_ cycle rate is termed bundle-sheath leakiness (Farquhar, 1983), or leakiness (*ϕ*), and is a key parameter of C_4_ photosynthesis.

Leakiness is generally quantified using a carbon isotope approach based on the model of carbon isotope discrimination (Δ) of C_4_ photosynthesis (Farquhar, 1983). The simplified version of the C_4_ discrimination model indicates that Δ is determined by *ϕ* and *C*_i_/*C*_a_, the ratio of intercellular CO_2_ concentration (*C*_i_) to atmospheric CO_2_ concentration (*C*_a_):

$$\Delta =a+(b_{\text{4}}+\phi \left(b_{\text{3}}-s\right)-a)C_{\text{i}}/C_{\text{a}},$$ (1)

with *a* the fractionation during diffusion of CO_2_ in air, *b*_4_ the combined fractionation of PEP carboxylation and the preceding fractionation associated with dissolution of CO_2_ and conversion to HCO_3_^−^, *b*_3_ the fractionation by Rubisco and *s* the fractionation during leakage of inorganic C out of the bundle sheath. Since leaf-level intrinsic water use efficiency (WUE_i_; net CO_2_ assimilation rate/stomatal conductance) is a function of *C*_i_ and *C*_a_, combined with Eqn 1 we have:

$${\text{WUE}}_{\text{i}}=\frac{C_{\text{a}}}{1.6}\left(1-\frac{\Delta -a}{b_{4}+\left(b_{3}-\text{s}\right)\phi -a}\right),$$ (2)

showing that WUE_i_ can be estimated from Δ if *ϕ* is known. Similar to the application of Δ to quantify WUE_i_ of C_3_ plants (Farquhar and Richards, 1984; Yang *et al.*, 2016*b*), Δ was suggested as a potentially promising screening tool for breeding and improvement of C_4_ crops (Henderson *et al.*, 1998; Gresset *et al.*, 2014; von Caemmerer *et al.*, 2014). However, such endeavours require knowledge of the magnitude and variability of *ϕ* and relevant environmental drivers. Early studies led to the notion that leakiness is relatively constant within a species with moderate short-term environmental variation, e.g. in response to light, CO_2_, and temperature (Henderson *et al.*, 1992). A constant leakiness would largely simplify the relationship between WUE_i_ and Δ. However, more recent studies suggest dynamic variation of leakiness in response to short-term variation of irradiation (Kromdijk *et al.*, 2010; Pengelly *et al.*, 2010; Ubierna *et al.*, 2013; Bellasio and Griffiths, 2014) or temperature (von Caemmerer *et al.*, 2014).

Analysis of dynamic changes of leakiness in response to short-term variation of atmospheric CO_2_ provides an opportunity for studying the functioning of the CCM. However, model analyses suggested an increase of leakiness with increasing *C*_i_ (von Caemmerer and Furbank, 1999; Kiirats *et al.*, 2002; Ghannoum, 2009; Yin *et al.*, 2011), while (the few) experimental studies using the carbon isotope approach showed no clear response of leakiness to *C*_i_ (Henderson *et al.*, 1992; Pengelly *et al.*, 2012).

Long-term effects of environmental drivers on leakiness have been observed. Thus, drought stress was shown to increase leakiness of several species (Saliendra *et al.*, 1996; Williams *et al.*, 2001; Fravolini *et al.*, 2002), and this effect was attributed to the relative activities of Rubisco/PEPc (Saliendra *et al.*, 1996). N stress was reported to affect leakiness of several sugarcane species (Meinzer and Zhu, 1998), but another study with different species found no effect of N fertilizer supply (Fravolini *et al.*, 2002). Increased CO_2_ concentration was shown to increase (Watling *et al.*, 2000; Fravolini *et al.*, 2002) or have no effect (Williams *et al.*, 2001) on leakiness. Currently, it is unknown if vapour pressure deficit (VPD) during plant growth has an effect on leakiness. Studies on long-term effects of N nutrition, water supply, or CO_2_ concentration during growth on leakiness using biomass-based Δ have considerable uncertainty related to post-photosynthetic fractionation and the temporal mismatch between biomass formation and gas exchange measurements as discussed by many authors (Henderson *et al.*, 1992; Cousins *et al.*, 2008; von Caemmerer *et al.*, 2014).

Scarcity of experimental evidence is partially due to the fact that quantifying leakiness using combined measurements of gas exchange and ∆ (‘on-line’ ∆) is a laborious task. Measurement artefacts, i.e. CO_2_ leak fluxes between the leaf cuvette and the surrounding air and isotopic disequilibria between photosynthetic and respiratory CO_2_ fluxes, can lead to errors of measured ∆ of several permil (Gong *et al.*, 2015). This technical issue is particularly relevant for C_4_ species, as the ∆ of C_4_ plants is generally much lower than that of C_3_ plants (Farquhar, 1983).

A previous study in our lab demonstrated that *Cleistogenes squarrosa* (Trin.) Keng—a perennial C_4_ (NAD-ME) grass and co-dominant species in the semiarid steppe of Inner Mongolia—had a large and variable biomass-based Δ of leaves (6–9‰; Yang *et al.*, 2011), potentially indicating a high and variable *ϕ*. As *ϕ* is related to WUE_i_ (Eqn 2) and potentially driven by environmental factors, we were interested to unravel the mechanisms governing Δ in this C_4_ species. Furthermore, understanding the physiological response of *C. squarrosa* to N nutrition and VPD may provide new insight into the adaptation of this species to its habitat given that: (i) water and N availability are the major limiting factors that determine primary production and resource use efficiency of plants in the semiarid steppes of Inner Monglia (Gong *et al.*, 2011) and (ii) the abundance of C_4_ plants has increased substantially during the past several decades in the Mongolian plateau (Wittmer *et al.*, 2010). Given the lack of knowledge on long-term effects of VPD or N nutrition and short-term dynamic effects of CO_2_ on *ϕ*, we performed controlled environment experiments with *C. squarrosa* grown under a low or high level of VPD combined with a low or high level of N fertilizer supply. Leakiness was determined using combined measurements of gas exchange and ∆ (on-line ∆) following the protocols of Gong *et al.* (2015).

## Materials and methods

### Plant material and growth conditions

*Cleistogenes squarrosa* is an NAD-ME type (determined by ^14^C labelling) C_4_ grass, with a ‘chloridoid’ type of Kranz anatomy (Pyankov *et al.*, 2000). Stands of *C. squarrosa* were grown from seed in plastic pots (5 cm diameter, 35 cm depth) filled with quartz sand. Pots were placed in growth chambers (PGR15, Conviron, Winnipeg, Canada) at a density of 236 plants m^−2^. A photosynthetic photon flux density (PPFD) of 800 µmol m^−2^ s^−1^, provided by cool white fluorescent tubes, was maintained at the top of the canopies during the 16 h-long photoperiods. Air temperature was maintained at 25 ºC during photo- and dark periods. A CO_2_ concentration ([CO_2_]) of 386±3 µmol mol^−1^ (mean±SD, *n*>400) in chamber air was maintained during the photoperiods (cf. Schnyder *et al.*, 2003). A modified Hoagland nutrient solution was supplied three times per day by an automatic irrigation system throughout the entire experiment similar to Lehmeier *et al.* (2008). The time of first watering is referred to as imbibition of seeds. Before germination, all chambers were run with a VPD of 0.63 kPa (corresponding to a relative humidity (RH) of 80%).

### N nutrition and VPD treatments

The study had a 2 × 2 factorial design, with N fertilizer supply and VPD as factors, two levels (low and high, see below) for each factor, and four replicates (four replicate stands) for each combination of N and VPD level. About 1 week after the imbibition of seeds (i.e. first watering), VPD and N treatment were implemented until the end of the experiment. The nutrient solution with 7.5 mM N in the form of equimolar concentrations of calcium nitrate and potassium nitrate was supplied to chambers of the low N treatment (N1), while another nutrient solution with 22.5 mM N was supplied to chambers of the high N treatment (N2). The concentration of other nutrients was the same in both nutrient solutions: 1.0 mM MgSO_4_, 0.5 mM KH_2_PO_4_, 1 mM NaCl, 125 µM Fe-EDTA, 46 µM H_3_BO_3_, 9 µM MnSO_4_, 1 µM ZnSO_4_, 0.3 µM CuSO_4_, 0.1 µM Na_2_MoO_4_. VPD (difference between actual vapour pressure and the saturation vapour pressure) was maintained at 0.63 kPa (corresponding to an RH of 80%) in the chambers of the low VPD treatment (V1) or at 1.58 kPa (corresponding to an RH of 50%) in the chambers of the high VPD treatment (V2). Each chamber (or each stand) was assigned to one of the treatments: N1V1, N1V2, N2V1, and N2V2. As we had a total of four chambers, in order to repeat each treatment four times, all replicates were accommodated in four sequential experimental runs (in total, 16 stands were grown) (cf. Table S1 in Liu *et al.* 2016). In each treatment, two replicate chambers were supplied with CO_2_ from a mineral source (δ ^13^C_CO2_ of −6‰) and the other two chambers with CO_2_ from an organic source (δ ^13^C_CO2_ of −33‰). Carbon isotope discrimination by the canopies led to some ^13^C-enrichment of the CO_2_ inside the growth chambers. However, that effect was small due to a high rate of air flow through the chambers: quasi-continuous on-line measurements of δ ^13^C_CO2_ inside the growth chambers demonstrated a δ ^13^C_CO2_ inside the chambers with the ^13^C-enriched CO_2_ source of −5.2±0.1‰ (mean±SD, *n*=23) and −31.7±0.1‰ (*n*=23) inside the chambers with the ^13^C-depleted CO_2_.

### Gas exchange measurement system

The method reported by Gong *et al.* (2015) was used for leaf-level combined measurements of gas exchange and ^13^C discrimination (on-line ∆). Measurements were performed using a leaf-level ^13^CO_2_/^12^CO_2_ gas exchange and labelling system, which included a portable CO_2_ exchange system (LI-6400, LI-COR Inc., Lincoln, NE, USA) housed in a gas exchange mesocosm (Gong *et al.*, 2015). The air supply to the mesocosm and LI-6400 was mixed from CO_2_-free, dry air, and CO_2_ of know δ^13^C_CO2_ (cf. Schnyder *et al.*, 2003). [CO_2_] inside the mesocosm (growth chamber) was monitored with an infrared gas analyser (LI-6262, LI-COR Inc.). During the measurement, the plant to be measured and the sensor head of the LI-6400 were placed inside the mesocosm, with concentration and δ^13^C of CO_2_ in both gas exchange facilities monitored and controlled. Using this set-up, we separately controlled the concentration and δ^13^C in both facilities (sensor head and growth chamber) for purposes of gas exchange measurement or ^13^C labelling (see below).

The mesocosm and leaf cuvette systems were coupled to a continuous-flow isotope ratio mass spectrometer (IRMS; Delta^plus^ Advantage equipped with GasBench II, ThermoFinnigan, Bremen, Germany) via a stainless steel capillary. Sample air was drawn through the capillary via a peristaltic pump and passed through a 0.25 ml sample loop attached to the 8-port Valco valve of the GasBench II. Sample air in the loop was introduced into the IRMS via an open split after passage of a dryer (Nafion) and a GC column (25 m×0.32 mm Poraplot Q; Chrompack, Middleburg, The Netherlands). After every second sample a VPDB-gauged CO_2_ reference gas was injected into the IRMS via the Open Split. The whole-system precision (SD) of repeated measurements was 0.10‰ (*n*=50). For further details of the method see Gong *et al.* (2015).

### Gas exchange and concurrent on-line ^13^C discrimination measurements

Gas exchange and on-line ∆ measurements followed the optimized protocols suggested by Gong *et al.* (2015). In order to minimize the effects of any CO_2_ leak artefact across the gaskets of the clamp-on leaf cuvette and the surrounding (mesocosm) air, [CO_2_] inside the mesocosm was maintained close to that of the leaf cuvette of the LI-6400 (with the difference <30 μmol mol^−1^), and the same CO_2_ source was supplied to the mesocosm and LI-6400. By these means, the diffusive gradient of ^13^CO_2_ or ^12^CO_2_ between leaf cuvette and the surrounding air was effectively minimized (Gong *et al.*, 2015). In order to also minimize the isotopic disequilibrium artefact between the photosynthetic and respiratory CO_2_ flux, the same CO_2_ source was used for growing plants and for on-line leaf-level ∆ measurements (Gong *et al.*, 2015).

Gas exchange and on-line ∆ measurements were performed on the fully expanded young leaves near the tip of major tillers (the second youngest fully expanded leaf), started at about 38 days and ended at about 53 days after the imbibition of seeds. We used the standard 2 × 3 cm cuvette of LI-6400. Two leaf blades from separate major tillers of the same plant were carefully placed in the cuvette. This was done to compensate for the narrow leaf width of *C. squarrosa* (about 3 mm). The enclosed leaf area was determined after gas exchange measurements by photographing the leaf blades and analysis with ImageJ software (National Institutes of Health, Bethesda, MD, USA). All gas exchange parameters were recomputed using the measured enclosed leaf area.

Photosynthetic CO_2_ response curves were taken with the successive CO_2_ steps of 390, 800, 390, 260, 180, 120, 90, 60, and 30 μmol mol^−1^ ([CO_2_] in the cuvette) on at least two plants randomly chosen from each growth chamber. In total, 42 plants were measured. The data of five plants were eliminated from further analysis due to extremely low leaf N contents. Finally, each treatment had nine to ten replicates of individual plants. During the gas exchange measurements, the environmental parameters were maintained close to the growth conditions: leaf temperature was maintained at 25 °C, PPFD at 800 µmol m^−2^ s^−1^, leaf-to-air VPD at 0.76–0.96 kPa (V1 treatment) or 1.4–1.6 kPa (V2 treatment; Supplementary Fig. S1 at *JXB* online) at all CO_2_ levels. To generate the appropriate VPD for gas exchange measurements, the inflow air stream (well-mixed dry air) was passed through a gas washing bottle placed in a temperature-controlled water bath. For each step of the CO_2_ response, the [CO_2_] in the mesocosm was also adjusted to be close to that of the cuvette. Leaves were acclimated for at least 30 min before the start of gas exchange measurements. After CO_2_ exchange rates reached a steady state at each step of the CO_2_ response, gas exchange parameters (measured by LI-6400), e.g. net CO_2_ assimilation rate (*A*), transpiration rate (*E*), and stomatal conductance to vapour (*g*_s_) were recorded. Instantaneous water use efficiency was calculated as WUE_ins_=*A*/*E*; intrinsic water use efficiency was calculated as WUE_i_=*A*/*g*_s_.

Among the 37 CO_2_ response curves, 22 plants were concurrently measured for on-line ^13^C discrimination. For this, the [CO_2_] and δ^13^C of the incoming (*C*_in_ and δ_in_) and outgoing cuvette air (*C*_out_ and δ_out_) were measured on five to six plants per treatment combination. All measurements of δ^13^C were corrected for the non-linearity effect of the IRMS (Elsig and Leuenberger, 2010). ^13^C discrimination during net CO_2_ assimilation (∆) was calculated according to Evans *et al.* (1986):

$$\Delta =\frac{\xi (\delta_{out}-\delta_{in})}{1+\delta_{out}-\xi (\delta_{out}-\delta_{in})},$$ (3)

where *ξ=C*_in_/(*C*_in_−*C*_out_).

### Determination of leaf respiration rate in light

Additional measurements were performed to determine leaf respiration rate in light (*R*_L_). This was done with leaves of the same age class as used for CO_2_ response measurements using the ^13^C labelling method reported by Gong *et al.* (2015). All measurements were performed under growth conditions (i.e. at *C*_a_ of 390 μmol mol^−1^, PPFD of 800 µmol m^−2^ s^−1^). Briefly, ∆ of each leaf was measured first with the same CO_2_ source as used for growing plants, then the same measurement was repeated with the other CO_2_ source (i.e. the ^13^C-enriched or -depleted CO_2_ source). Making use of isotopic disequilibria between photosynthetic and respiratory CO_2_ fluxes derived from the two sets of on-line ∆ measurements, the ratio of *R*_L_ to net CO_2_ assimilation rate (*A*) was solved as:

$$R_{\text{L}}/A=\left(\delta_{\text{AE}}-\delta_{\text{AD}}\right)/\left(\delta_{\text{outE}}-\delta_{\text{outD}}\right)-\text{1},$$ (4)

$$\delta_{\text{A}}=\left(\delta_{\text{in}}C_{\text{in}}-\delta_{\text{out}}C_{\text{out}}\right)/\left(C_{\text{in}}-C_{\text{out}}\right),$$ (5)

where *δ*_A_ and *δ*_out_ are the δ^13^C of net CO_2_ assimilation and that of CO_2_ in the cuvette, respectively, and subscripts E and D indicate the relevant parameters measured with the ^13^C-enriched or the ^13^C-depleted sources of CO_2_, respectively. *R*_L_ was measured on six individuals in each treatment. *R*_L_ of other leaves of the same treatment was estimated by multiplying *A* of that leaf by the treatment-specific mean of *R*_L_/*A*. For further details of the methodology see Gong *et al.* (2015).

### Bundle-sheath leakiness (ϕ), C_3_ cycle rate and C_4_ cycle rate

Bundle-sheath leakiness was determined using the complete version of the Farquhar C_4_ discrimination model (Farquhar, 1983), modified to include the ternary correction (Farquhar and Cernusak, 2012):

$$\begin{array}{l} \Delta =\frac{1}{1-t}[a_{\text{b}}\frac{C_{\text{a}}-C_{\text{L}}}{C_{\text{a}}}+a\frac{C_{\text{L}}-C_{\text{i}}}{C_{\text{a}}}]+\frac{1+t}{1-t}[\left(e_{\text{s}}+a_{\text{l}}\right)\frac{C_{\text{i}}-C_{\text{m}}}{C_{\text{a}}}\\ \;+\frac{b_{4}+\phi \left(\frac{b_{3}C_{\text{bs}}}{C_{\text{bs}}-C_{\text{m}}}-s\right)}{1+\frac{\phi C_{\text{m}}}{C_{\text{bs}}-C_{\text{m}}}}\frac{C_{\text{m}}}{C_{\text{a}}}], \end{array}$$ (6)

where *C*_a_, *C*_L_, *C*_i_, *C*_m_, and *C*_bs_ are the CO_2_ concentrations in air (*C*_a_=*C*_out_ during gas exchange measurements), at the leaf surface, in the intercellular airspace, in the mesophyll cell, and in the bundle-sheath cell, respectively. *C*_L_ was calculated as *C*_L_=*C*_a_−1.37*A*/*g*_BL_, with *g*_BL_ the boundary layer conductance to vapour calculated by the LI-6400 software. *a*_b_=2.9‰, *a=*4.4‰, *e*_s_=1.1‰, *a*_l_=0.7‰, and *s*=1.8‰ denote fractionation constants associated with CO_2_ diffusion across boundary layer, across stomata, dissolution in water, diffusion in liquid phase, and leaking out of bundle sheath cells, respectively. *t* represents the ternary correction factor (Farquhar and Cernusak, 2012):

$$t=\left(\text{1}+\;\bar{a}\right)E/\left(\text{2}g_{\text{sc}}\right),$$ (7)

where *E* is the transpiration rate and *g*_sc_ is the total conductance to CO_2_: *g*_sc_=1/(1.6/*g*_s_+1.37/*g*_BL_), where *g*_s_ is the stomatal conductance to vapour. *ā* is the weighted fractionation across boundary layer (*a*_b_=2.9‰) and stomata (*a*=4.4‰): *ā=*[*a*_b_(*C*_a_−*C*_L_)+*a*(*C*_L_−*C*_i_)]/(*C*_a_−*C*_i_). *b*_3_ (^13^C fractionation during carboxylation by Rubisco including respiratory fractionation) and *b*_4_ (^13^C fractionation by CO_2_ dissolution, hydration, and PEPc carboxylation including respiratory fractionation) were calculated according to Farquhar (1983):

$$b_{\text{3}}=b\prime_{\text{3}}-eR_{\text{L}}/V_{\text{c}}-0.\text{5}fV_{\text{o}}/V_{\text{c}},\text{ and}$$ (8)

$$b_{\text{4}}=b\prime_{\text{4}}-0.\text{5}eR_{\text{L}}\left(\text{1}-\phi \right)/\left(A+0.\text{5}R_{\text{L}}\right),$$ (9)

where *b*′_3_=30‰, *b*′_4_=–5.7‰, *e*=–6‰ (Ghashghaie *et al.*, 2003; Kromdijk *et al.*, 2010), *f*=11.6‰ (Ghashghaie *et al.*, 2003; Lanigan *et al.*, 2008), and *V*_c_ is the rate of Rubisco carboxylation and *V*_o_ is the rate of oxygenation. In our experiments, the same source CO_2_ was used for growing plants and for online ∆ measurements; therefore an additional term (cf. *e*′ in Ubierna *et al.* 2013) accounting for the discrepancy of δ^13^C_CO2_ between growth environment and gas exchange measurements was not included. Here, it is assumed that respiration in mesophyll cells (*R*_m_) and bundle-sheath cells (*R*_bs_) both equal 0.5*R*_L_ (von Caemmerer and Furbank, 1999; Pengelly *et al.*, 2010).

To solve *ϕ* using Eqns 7–9, *C*_m_, *C*_bs_, *V*_o_, and *V*_c_ must be estimated. Those parameters were determined using the equations of the C_4_ photosynthesis model of von Caemmerer and Furbank (1999). *C*_m_ can be calculated from:

$$g_{\text{m}}=A/\left(C_{\text{i}}-C_{\text{m}}\right),$$ (10)

if mesophyll conductance to CO_2_ is known. It has been generally assumed that *g*_m_ is high and non-limiting for CO_2_ assimilation in C_4_ photosynthesis. Thus, in many published works on *ϕ*, *g*_m_ was assumed to be infinite, i.e. *C*_i_=*C*_m_. However, a recent study using a new oxygen isotope approach found that *g*_m_ of C_4_ species ranged between 0.66 and 1.8 mol m^−2^ s^−1^ measured at a leaf temperature of about 32 °C (Barbour *et al.*, 2016). We calculated the minimum *g*_m_ as: *g*_mmin_=*A*/*C*_i_ (von Caemmerer *et al.*, 2014). These results showed that *g*_mmin_ decreased with increasing *C*_i_ (see Supplementary Fig. S2). Further, we assumed that the young leaves of *C. squarrosa* had a *g*_m_ of 0.66 mol m^−2^ s^−1^ at the growth *C*_a_ of 390 μmol mol^−1^. Appling a constant ratio of *g*_m_/*g*_mmin_ across the CO_2_ response curve, we estimated the response of *g*_m_ to *C*_i_. The estimates of *g*_m_ ranged between 0.2 and 2.4 mol m^−2^ s^−1^ and decreased with increasing *C*_i_ (Supplementary Fig. S2); similarly, decrease of *g*_m_ with increasing *C*_i_ was found in other C_4_ species using the oxygen isotope approach (Osborn *et al.*, 2016) or an ‘*in vitro V*_pmax_’ method (N. Ubierna, personal communication).

A simplified version of the C_4_ photosynthesis model for enzyme-limited CO_2_ assimilation rate (von Caemmerer and Furbank, 1999) can be used to model the photosynthetic CO_2_ response curve: *A*=min[(*V*_p_−*R*_m_+*g*_bs_*C*_m_), (*V*_cmax_−*R*_L_)], where *V*_P_ is the rate of PEP carboxylation, *V*_cmax_ is the maximum rate of Rubisco carboxylation, and *g*_bs_ is the bundle-sheath conductance to CO_2_. *A* is determined by the term *V*_cmax_−*R*_L_ at high CO_2_ concentrations. According to this model, we assumed that *V*_cmax_=*A*_max_+*R*_L_, where *A*_max_ is the maximum net assimilation rate observed in the photosynthetic light response curves measured on leaves of the same age at ambient CO_2_ (data shown in Supplementary Fig. S3). Treatment-specific *V*_cmax_ of *C. squarrosa* ranged between 16 and 19 μmol m^−2^ s^−1^, which is in the range of *V*_cmax_ of C_4_ grass used in Earth System Models (13–33 μmol m^−2^ s^−1^; Rogers, 2014). *C*_bs_ was estimated using equations of von Caemmerer and Furbank (1999):

$$C_{\text{bs}}=\frac{\gamma^{*}O_{\text{bs}}+K_{\text{c}}(1+\frac{O_{\text{bs}}}{K_{\text{o}}})(A+R_{\text{L}})/V_{\text{cmax}}}{1-(A+R_{\text{L}})/V_{\text{cmax}}},$$ (11)

$$O_{\text{bs}}=\alpha A/\left(0.0\text{47}g_{\text{bs}}\right)+O_{\text{m}},$$ (12)

where *O*_bs_ is the bundle-sheath oxygen concentration, and *O*_m_ (210 mmol mol^−1^) is the oxygen concentration in mesophyll cells; γ* (0.000193) is half of the reciprocal of Rubisco specificity; *K*_c_ and *K*_o_ are the Michaelis–Menten constants of Rubisco for CO_2_ (650 μmol mol^−1^) and O_2_ (450 μmol mol^−1^) at 25 °C, respectively; *α* (0.1) is the fraction of PSII activity in the bundle-sheath; *g*_bs_=0.003 mol m^−2^ s^−1^. Furthermore, *V*_o_/*V*_c_ was calculated as *V*_o_/*V*_c_=2γ*O_bs_/*C*_bs_. Knowing *V*_o_/*V*_c_, *V*_o_ and *V*_c_ were calculated using the equation: *V*_c_=*A*+*R*_L_+0.5*V*_o_. The values of model parameters and equations were derived from von Caemmerer and Furbank (1999). Knowing *ϕ* and applying the assumption that *R*_m_=*R*_bs_=0.5*R*_L_ (von Caemmerer and Furbank, 1999; Pengelly *et al.*, 2010), the rate of PEP carboxylation (*V*_p_) or Rubisco carboxylation (*V*_c_) was estimated (see Supplementary Fig. S4). As *C*_m_ and *C*_bs_ cannot be directly measured, the determination of both parameters in this study was associated with some uncertainty. Another commonly used assumption in calculating leakiness using the complete or simplified version of Eqn 6 is that CO_2_ is in equilibrium with HCO_3_^−^ in the mesophyll cytoplasm. The extent of this equilibrium is determined by the relative rate of PEP carboxylation and hydration of CO_2_ (*V*_h_). If CO_2_ is not in equilibrium with HCO_3_^−^, *b*′_4_ of –5.7‰ in Eqn 9 should be replaced by *b*′_4_=–5.7 + 7.9*V*_p_/*V*_h_ (Farquhar 1983). Thus, we calculated *ϕ* using C_4_ discrimination models with different assumptions on *g*_m_, *C*_bs_, and *V*_p_/*V*_h_ (Supplementary Fig. S5). These results are compared and discussed below.

### Fitting of CO_2_ response curves

For each plant, measured *A* was plotted against the respective intercellular CO_2_ concentration. A model of C_4_ photosynthesis was used to fit the CO_2_ response curves (Wang *et al.*, 2012):

$$A=a\prime (\text{1}-{\text{e}}^{-b}{}^{\prime x})+c\prime$$ (13)

where *x* is *C*_i_ and *c′* is *R*_L_. Using the fitted parameters (*a′* and *b′*) of each CO_2_ response curve, CO_2_-saturated net assimilation rate (*A*_sat_) was obtained as (*a′*+*c′*), and carboxylation efficiency (CE) was calculated as *b′*(*a′*+*c′*) (the initial slope of the *A*–*C*_i_ curve). For CO_2_ response curves of C_4_ leaves, *A*_sat_ is proportional to Rubisco activity, while CE is proportional to the PEP carboxylase activity (von Caemmerer and Furbank, 1999; Yin *et al.*, 2011). The ratio of *A*_sat_ to CE was calculated to indicate the ratio of Rubisco to PEPc activities.

### Leaf trait parameters and nitrogen nutrition index

N content in dry mass of leaves previously used for gas exchange measurements was measured using an elemental analyser (NA 1110, Carlo Erba, Milan, Italy). For sample preparation, leaves were dried at 60 °C for 48 h, weighed and then milled. N content on a mass (N_mass_, %DM) and leaf area basis (N_area_, g m^−2^) were calculated. Specific leaf area (SLA, cm^2^ mg^−1^) was obtained as SLA=leaf area/leaf dry mass. In each experiment, a set of individual plants (four plants per chamber) were sampled four or five times (twice per week) after canopy closure (when leaf area index was >2) for the determination of standing aboveground biomass, and N content in total aboveground dry mass. Those data were used to calculate nitrogen nutrition index (NNI) according to Lemaire *et al.* (2008).

### Statistical analysis

Two-way ANOVA was used to test the effects of N supply, VPD, and their interactions on leaf traits and gas exchange parameters, using general linear models of SAS (SAS 9.1, SAS Institute, USA). The effects of N nutrition and VPD on the relationship between ln*C*_i_ and leakiness were analysed using a dummy regression approach; the parallelism and coincidence of linear regressions of different treatments were tested according to Berenson *et al.* (1983). Non-linear regression analysis on photosynthetic CO_2_ response curve of individual plants was performed using the Nlin procedure of SAS.

## Results

### Leaf traits and photosynthetic CO_2_ assimilation

N supply increased N_mass_ of fully expanded young leaves by 25% (*P*<0.05) and also tended to increase N_area_ (*P*=0.06), but had no effect on SLA (Table 1). N supply also significantly increased nitrogen nutrition index (NNI; determined from N content and aboveground standing biomass of each stand): NNI was 0.88 ± 0.05 (mean±SD, *n*=4 replicate stands) for the N1V1 treatment, 0.71±0.06 for the N1V2 treatment, 1.25±0.06 for the N2V1 treatment, and 1.32±0.05 for the N2V2 treatment. The effects of VPD and the interaction of VPD and N supply on N_mass_, N_area_ and SLA were all non-significant (*P*>0.05) (Table 1).

**Table 1.** *Leaf trait and gas exchange parameters of* C. squarrosa *measured under the same environmental conditions as during growth (leaf temperature 25 °C, PPFD 800 μmol m*^*−2*^*s*^*−1*^*, [CO*_*2*_*] 390 μmol mol*^*−1*^*, VPD 0.8 kPa for V1 and 1.6 kPa for V2*) Plants were grown at low or high N fertilizer supply (N1 or N2) combined with low or high VPD (V1 or V2). Leaf trait parameters include N content per unit dry mass (N_mass_, % DM) and per leaf area (N_area_, g m^−2^), and specific leaf area (SLA, cm^2^ mg^−1^). Gas exchange parameters: net CO_2_ assimilation rate (*A*, μmol m^−2^ s^−1^), transpiration rate (*E*, mmol m^−2^ s^−1^), stomatal conductance to water vapor (*g*_s_, mol m^−2^ s^−1^), respiration rate in light (*R*_L_, μmol m^−2^ s^−1^), the ratio of internal to atmospheric CO_2_ concentration (*C*_i_/*C*_a_), instantaneous water use efficiency (WUE_ins_=*A*/*E*, mmol mol^−1^), and intrinsic water use efficiency (WUE_i_=*A*/*g*_*s*_, μmol mol^−1^). Data are shown as mean±SE (*n*=6 for *R*_L_, *n*=9–10 for the other parameters). Significant treatment effects (*P*<0.05) are shown in bold.

	N1		N2		*P*-value		
	V1	V2	V1	V2	N	VPD	N×VPD
N_mass_	2.4 ± 0.1	2.4 ± 0.1	3.0 ± 0.1	3.0 ± 0.2	**0.01**	0.81	0.79
N_area_	1.2 ± 0.2	1.3 ± 0.2	1.6 ± 0.2	1.6 ± 0.2	0.06	0.82	0.92
SLA	0.21 ± 0.02	0.20 ± 0.02	0.21 ± 0.02	0.21 ± 0.02	0.91	0.82	0.85
*A*	14.8 ± 0.9	13.1 ± 0.9	14.9 ± 0.9	12.3 ± 1.0	0.81	**0.03**	0.58
*E*	0.93 ± 0.08	1.14 ± 0.09	0.93 ± 0.08	1.23 ± 0.09	0.69	**0.01**	0.60
*g*_s_	0.10 ± 0.01	0.08 ± 0.01	0.11 ± 0.01	0.07 ± 0.01	0.69	**0.01**	0.45
*R*_L_	0.81 ± 0.26	0.62 ± 0.30	0.53 ± 0.10	0.51 ± 0.10	0.36	0.62	0.71
*C*_i_/*C*_a_	0.35 ± 0.03	0.26 ± 0.04	0.35 ± 0.03	0.25 ± 0.04	0.93	**0.01**	0.91
WUE_ins_	15.9 ± 0.6	11.7 ± 0.6	16.8 ± 0.6	10.0 ± 0.6	0.82	**0.01**	**0.04**
WUE_i_	153 ± 9	173 ± 9	154 ± 9	178 ± 10	0.79	**0.02**	0.82

The effects of N supply on N_mass_ and N_area_ were not associated with parallel effects on gas exchange parameters measured at growth CO_2_ level (390 μmol mol^−1^). Thus, N supply had no effect on *A*, *E*, *g*_s_, *C*_i_/*C*_a_, and WUE_ins_, and there was only a small interactive effect of N and VPD on WUE_i_ (Table 1). Conversely, VPD during growth had significant effects on all these parameters, but none of these effects demonstrated an interaction with N supply except for WUE_ins_. High VPD decreased *A* by 14%, *g*_s_ by 29%, *C*_i_/*C*_a_ by 27% at both low and high N, and WUE_ins_ by 26% at low N and 40% at high N. Conversely, high VPD increased *E* by 27% and WUE_i_ by 14% independently of N levels (Table 1). The rate of leaf respiration in light (*R*_L_) did not differ between treatments.

**Fig. 1.**
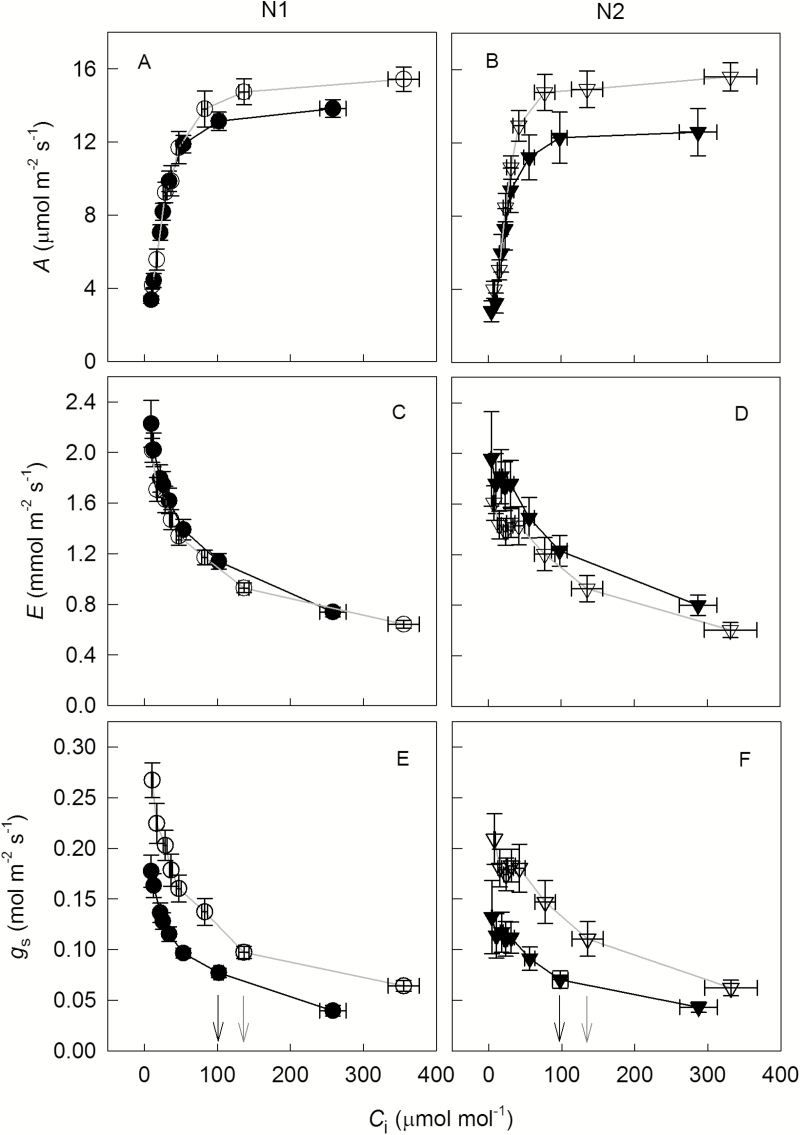
Net CO_2_ assimilation rate (*A*; A, B), transpiration rate (*E*; C, D) and stomatal conductance to water vapour (*g*_s_; E, F) in response to short-term variation of intercellular CO_2_ (*C*_i_) under low (N1, circles; A, C, E) or high N supply (N2, triangles; B, D, F) combined with low (V1, open symbols) or high VPD (V2, filled symbols). Data are shown as the mean±SE (*n*=9–10). Operating conditions of gas exchange measurements were the same as conditions in growth chambers (leaf temperature 25 °C, PPFD 800 μmol m^−2^ s^−1^, VPD 0.8 kPa for V1 and 1.6 kPa for V2). The corresponding *C*_i_ values at growth CO_2_ concentration (390 μmol mol^−1^) are indicated by grey (low VPD) and black arrows (high VPD) in (E) and (F).

The response of photosynthetic gas exchange parameters to dynamic changes of CO_2_ concentration (Fig. 1) showed typical CO_2_ responses of net CO_2_ assimilation rate. VPD had clear effects on gas exchange parameters: compared with low VPD, *A* was 20% lower (Fig. 1A, B), *E* was 21% higher (Fig. 1C, D), and *g*_s_ was 32% lower (Fig. 1E, F) at high VPD averaged over N levels and CO_2_ concentrations.

### Carbon isotope discrimination and bundle-sheath leakiness

**Fig. 2.**
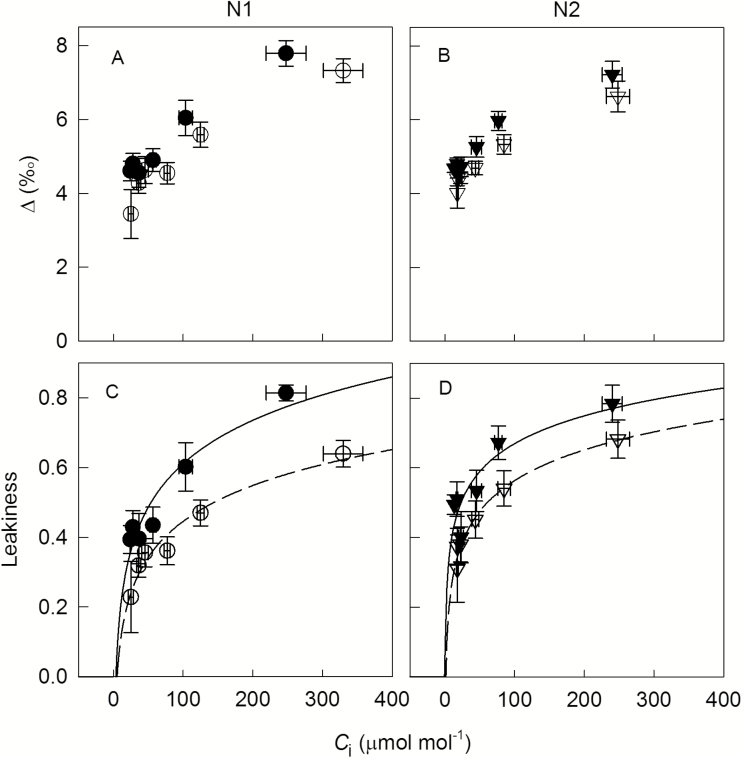
Carbon isotope discrimination (∆) during net CO_2_ exchange (A, B) and bundle sheath leakiness (C, D) of *Cleistogenes squarrosa* leaves in response to short-term variation of intercellular CO_2_ (*C*_i_). Plants were grown under low (N1, circles; A, C) or high N supply (N2, triangles; B, D) combined with low (V1, open symbols, dashed lines) or high VPD (V2, filled symbols, solid lines). Operating conditions of gas exchange measurements were the same as conditions in growth chambers (leaf temperature 25 °C, PPFD 800 μmol m^−2^ s^−1^, VPD 0.8 kPa for V1 and 1.6 kPa for V2). Data are shown as the mean±SE (*n*=5–6). The regressions were fitted using a function of *y*=*y*_0_+*a*ln(*x*); all regressions have *r*^2^>0.8.

**Fig. 3.**
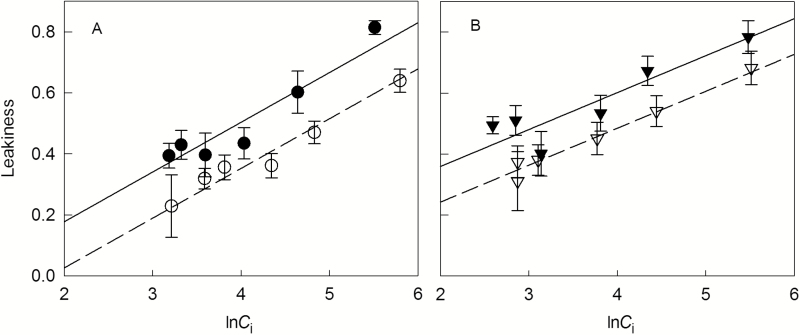
Relationships between bundle sheath leakiness and ln(*C*_i_) under low (N1, circles; A) or high N supply (N2, triangles; B) combined with low (V1, open symbols, dashed lines) or high VPD (V2, filled symbols, solid lines). Operating conditions of gas exchange measurements were the same as conditions in growth chambers (leaf temperature 25 °C, PPFD 800 μmol m^−2^ s^−1^, VPD 0.8 kPa for V1 and 1.6 kPa for V2). Data are shown as the mean±SE (*n*=5–6). The data of each N level were fitted by linear regression including a dummy variable indicating VPD treatments (*V*) after the confirmation of parallelism of the two regressions of VPD levels: with *V*=0 for low VPD and *V*=1 for high VPD (for equations see Table 2).

Carbon isotope discrimination (∆) during net CO_2_ exchange revealed strong dynamic responses to short-term variation of CO_2_ concentration; ∆ increased with increasing *C*_i_ in all treatments, and ranged from 4 to 8‰ (Fig. 2A, B). Compared at the same *C*_i_, ∆ was higher at high VPD than at low VPD. Estimates of *ϕ* also increased with increasing *C*_i_ in all treatments, and ranged from 0.23 to 0.81 (Fig. 2C, D). The relationship between *C*_i_ and *ϕ* followed a logarithmic function. Regression analyses between ln*C*_i_ and *ϕ* including VPD as a dummy variable indicated a significant VPD effect on *ϕ*: *ϕ* was higher by 0.13 at high VPD than at low VPD at both N levels (Fig. 3, Table 2). Plants grown with high N supply had a higher *ϕ* at moderate to low *C*_i_ (*C*_a_≤390) than plants with low N supply (Fig. 3). Accordingly, the slope of the linear regression between ln*C*_i_ and *ϕ* was significantly lower for the high N treatment than for the low N treatment (Table 2). The increase of *ϕ* along the gradient of *C*_i_ was apparently related to the discrepancy between rates of C_3_ and C_4_ cycles: *V*_c_ already reached the maximum at a *C*_i_<100 μmol mol^−1^, while *V*_p_ increased throughout the range of measured *C*_i_ (Supplementary Fig. S4). We also calculated *ϕ* using C_4_ discrimination models with different assumptions on *g*_m_, *C*_bs_, and *V*_p_/*V*_h_, and our conclusions on short-term response to CO_2_ and long-term response to VPD and N were not altered (see Supplementary Fig. S5).

**Table 2.** *Results of regression analysis on the relationship between* C_*i*_*and leakiness (ϕ*) The data for each N level were fitted by linear regression including a dummy variable indicating VPD treatments (*V*) after the confirmation of parallelism of the two regressions of VPD levels, with *V*=0 for low VPD and *V*=1 for high VPD; 95% confidence intervals of slope and coefficient of dummy variable (*V*) are shown. The coefficient of *V* quantifies the mean difference in *ϕ* between high and low VPD treatments across *C*_i_ levels. Both regressions are significant (*P*<0.001).

N level	equation	*r* ^2^	95% CI slope	95% CI coeff. *V*
N1	* ϕ *=−0.30+0.16ln*C*_i_+0.15*V*	0.94	0.13~0.20	0.09~0.21
N2	* ϕ *=0.001+0.12ln*C*_i_+0.12*V*	0.91	0.09~0.15	0.05~0.18

### Relationships between leaf traits and gas exchange parameters

**Fig. 4.**
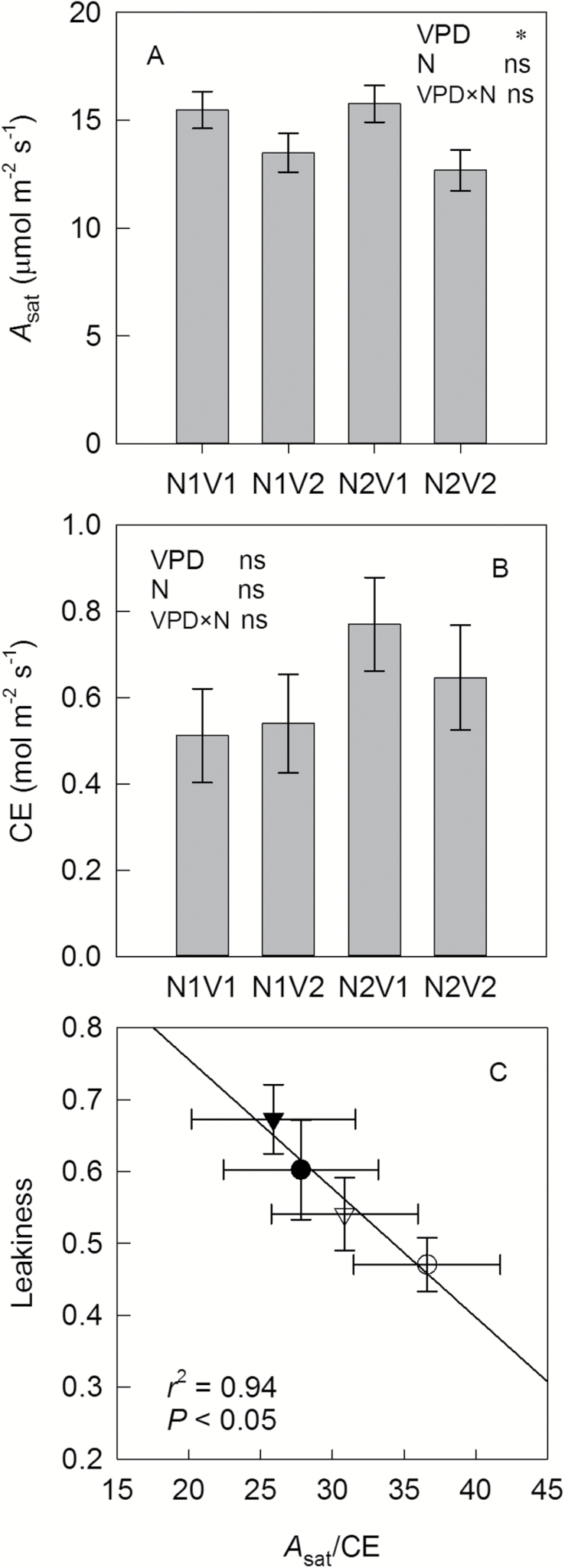
*A*_sat_ (A), carboxylation efficiency (CE; B) and the relationship between leakiness measured under growth conditions (390 μmol mol^−1^) and *A*_sat_/CE (μmol mol^−1^; C) under low or high N fertilizer supply (N1, circles; or N2, triangles) combined with low or high VPD (V1, open symbols; or V2, filled symbols). Data are shown as the mean±SE (*n*=9–10 for *A*_sat_ and CE, *n*=5–6 for leakiness). * indicates the treatment effect was significant at the *P*-level of 0.05, while ns indicates no significant effect.

**Fig. 5.**
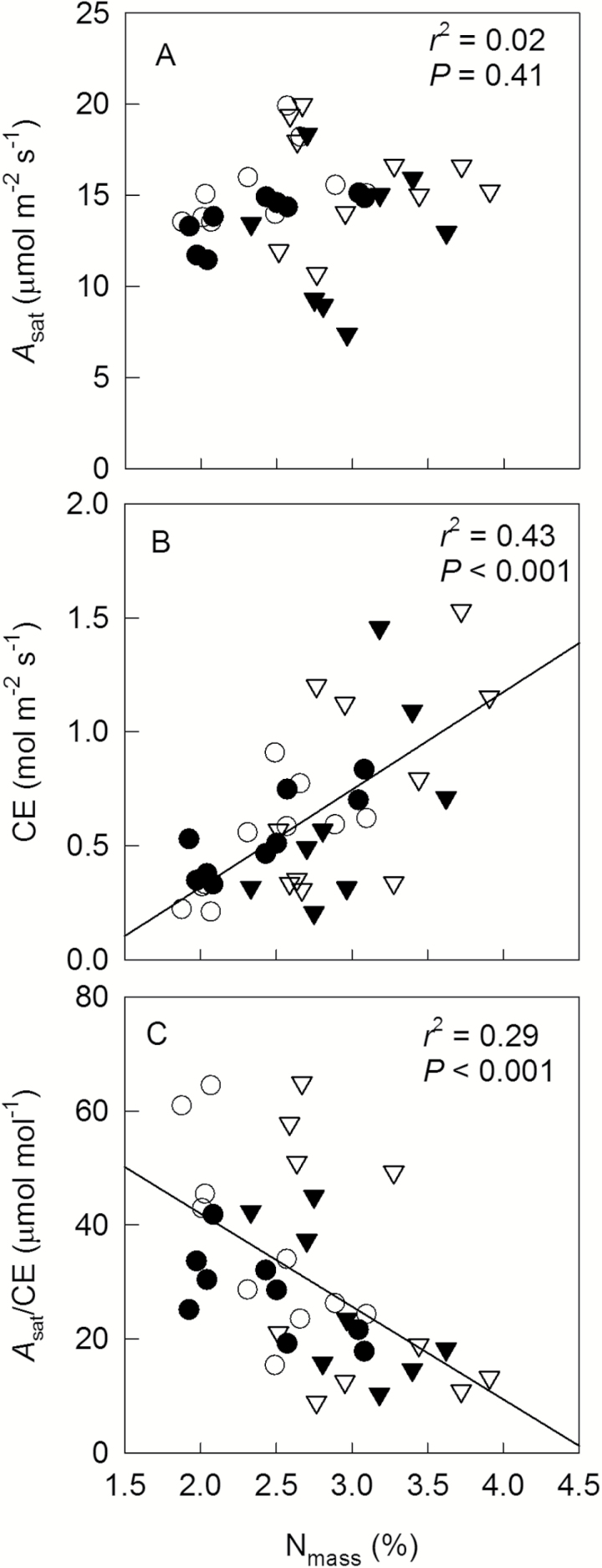
Correlations between N_mass_ and *A*_sat_ (A), CE (B) and *A*_sat_/CE (C) of leaves grown at low or high N fertilizer supply (N1, circles; or N2, triangles) combined with low or high VPD (V1, open symbols; or V2, filled symbols). Each symbol represents a data point of an individual plant.

**Fig. 6.**
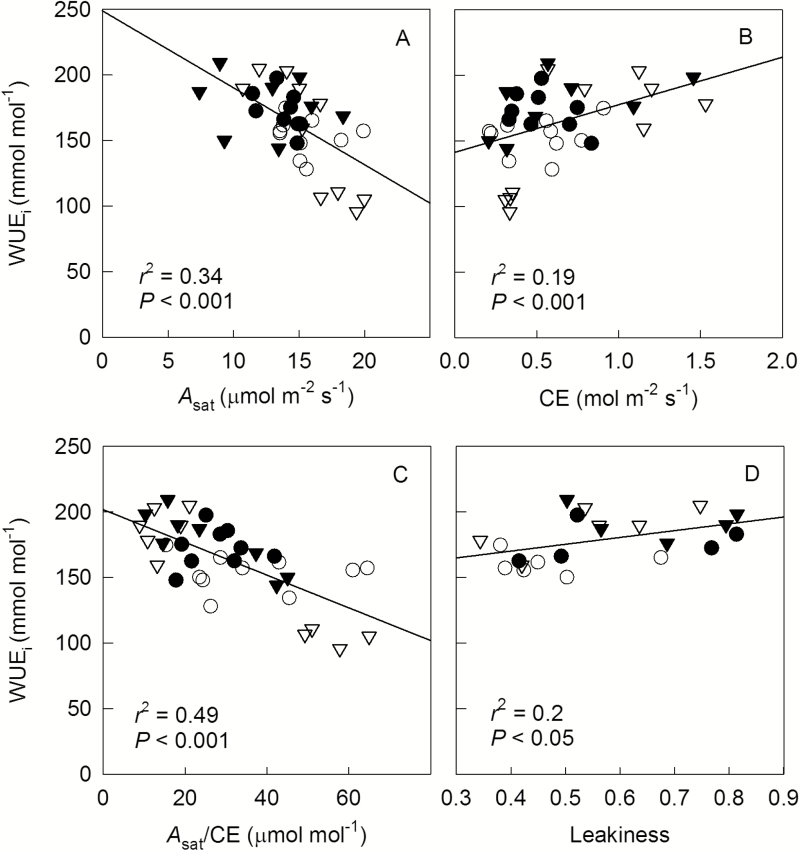
Correlations between WUE_i_ and *A*_sat_ (A), CE (B), *A*_sat_/CE (C), and leakiness (D) of leaves grown at low or high N fertilizer supply (N1, circles; or N2, triangles) combined with low or high VPD (V1, open symbols; or V2, filled symbols). Each symbol represents a data point of an individual plant.

VPD had a clear effect on *A*_sat_ (Fig. 4A): averaged over N treatments, high VPD caused a 15% reduction of *A*_sat_. N supply and N×VPD interactions had no effect on *A*_sat_. CE of leaves seemed to be slightly higher at high (0.71 mol m^−2^ s^−1^) than low N (0.53 mol m^−2^ s^−1^), although this effect was only significant at *P*=0.10 (Fig. 4B). *A*_sat_/CE was calculated to indicate the ratio of Rubisco to PEPc activities. Treatment-specific mean *A*_sat_/CE was negatively correlated with *ϕ* (*P*<0.05, Fig. 4C). Across all treatments, we found no correlation between *A*_sat_ and N_mass_ (*P*=0.41, Fig. 5A), but a positive correlation between CE and N_mass_ (*r*^2^=0.43, *P*<0.001, Fig. 5B) and a negative correlation between *A*_sat_/CE and N_mass_ (*r*^2^=0.29, *P*<0.001, Fig. 5C). WUE_i_ was negatively correlated with *A*_sat_ (*r*^2^=0.34, *P*<0.001, Fig. 6A) and *A*_sat_/CE (*r*^2^=0.49, *P*<0.001, Fig. 6C), but positively with CE (*r*^2^=0.19, *P*<0.001, Fig. 6B) and leakiness (*r*^2^=0.19, *P*<0.05, Fig. 6D). Furthermore, a positive correlation between WUE_i_ and leakiness was also found during short-term response to CO_2_ (*r*^2^>0.9, *P*<0.01, Supplementary Fig. S6).

## Discussion

This study indicates an effect of VPD of the growth environment on bundle-sheath leakiness: *ϕ* was higher at high VPD than at low VPD with an absolute difference of 0.13 at both N supply levels. This effect appeared to be constitutive, as it was manifest throughout a wide range of short-term variations of CO_2_ concentration. Remarkably, the higher leakiness at high VPD was associated with a 14% improved WUE_i_. Also, we observed an N effect on *ϕ* that responded to short-term variation of CO_2_ levels: high N supply increased *ϕ* of leaves at ambient to low CO_2_ levels (*C*_a_≤390 μmol mol^−1^). Further, *ϕ* responded dynamically to short-term changes of [CO_2_], in support of theoretical predictions of C_4_ photosynthesis models (von Caemmerer and Furbank, 1999; Yin *et al.*, 2011). Lastly, across treatments, a positive correlation between WUE_i_ and leakiness was evident, pointing to a trade-off between WUE_i_ and energy use efficiency of this species.

Unfortunately, leakiness cannot be measured directly, and the online ∆^13^C approach for estimations of leakiness in conjunction with the C_4_ photosynthetic discrimination model of Farquhar (1983) can lead to inaccurate estimates of leakiness if improper simplifications are made (Ubierna *et al.*, 2011, 2013; Gong *et al.*, 2015). One commonly used assumption is that *g*_m_ of C_4_ plants is infinite, and thus *C*_i_=*C*_m_. However, a recent study indicated that *g*_m_ of C_4_ plants is finite, although close to the high-end values of *g*_m_ of C_3_ plants (Barbour *et al.*, 2016). In our study, assuming a lower range of *g*_m_ across *C*_i_ levels (see Supplementary Fig. S5), or a constant *g*_m_ of 1.8 mol m^−2^ s^−1^ (data not shown) or an infinite *g*_m_ (Supplementary Fig. S5) did not modify observed treatment effects. Only if *g*_m_ was much lower at low compared with high VPD (e.g. *g*_m_ at N1V1 was 60% lower than at N1V2 treatment), would VPD effects on leakiness disappear. However, it is unlikely that *C. squarrosa* had lower *g*_m_ at low than at high VPD, as studies with C_3_ species generally showed a decrease of *g*_m_ by high VPD or water stress (Flexas *et al.*, 2008). Thus, our estimates of treatment effects on leakiness appeared to be largely insensitive to assumptions on *g*_m_.

Many studies using Eqn 6 have assumed that CO_2_ is in equilibrium with HCO_3_^−^ in the mesophyll cytoplasm. However, according to Cousins *et al.* (2008) that assumption might not be proper, especially for C_4_ grasses, which have lower carbonic anhydrase (CA) activity than C_4_ dicots. Our observations with the same species in the same experiment suggested that, under growth conditions, assimilated CO_2_ was in equilibrium with HCO_3_^−^, as indicated by the fact that δ^18^O of leaf cellulose did not differ between plants growing in the presence of CO_2_ with contrasting δ^18^O (Liu *et al.*, 2016). Thus the observed treatment effects on leakiness of *C. squarrosa* seemed not to be related to CA activity. Further, we calculated leakiness using different assumptions on *V*_p_/*V*_h_: *V*_p_/*V*_h_=0 or *V*_p_/*V*_h_=0.23 (mean of NAD-ME grasses in Cousins *et al.*, 2008) and observed a minor effect on leakiness (less than 0.06). Thus, the short-term response of leakiness to CO_2_ was not explained by changes in *V*_p_/*V*_h_. Applying another commonly used simplification that *C*_bs_ is sufficiently higher than *C*_m_, so that *C*_bs_/(*C*_bs_−*C*_m_)=1 and *C*_m_/(*C*_bs_−*C*_m_)=0, also had no influence on our conclusions on effects of environmental factors on leakiness.

### Effects of VPD and N nutrition on bundle-sheath leakiness

The study demonstrated a higher leakiness of plants grown at high VPD than at low VPD, and this effect was not modified by N nutrition. The present effect was not related to drought stress, as the elongation rate of phytomers ( Yang *et al.*, 2016*a*) and the growth rate of individuals (Liu *et al.*, 2016) were not influenced by VPD. The long-term response of leakiness to VPD was related to the decrease of *C*_i_/*C*_a_ and the increase of ∆ (0.55‰ at ambient CO_2_ level) at high VPD, although the direct and indirect influences cannot be clearly distinguished due to the intrinsic correlation between *C*_i_/*C*_a_ and ∆. The latter observation was also supported by the ^13^C discrimination of leaf dry mass, which was significantly higher at high VPD (5.0±0.2‰, averaged over N levels) than at low VPD treatment (4.4±0.2‰). Likely, the higher leakiness at high VPD resulted from an enhanced capacity of the C_4_ cycle relative to the C_3_ cycle at low *g*_s_, as was supported by the lower *A*_sat_/CE ratio and the lower *C*_i_/*C*_a_ at high VPD relative to low VPD. This interpretation is in line with the findings that the relative activities of Rubisco/PEPc decreased at low *g*_s_ under water stress, with the adjustment achieved by increasing activity of PEPc (Saliendra *et al.*, 1996; Foyer *et al.*, 1998) or decreasing sensitivity of PEPc to the inhibitor malate (Foyer *et al.*, 1998). Also, the data may shed light on the mechanism that underlies the increased leakiness under limited water supply (Saliendra *et al.*, 1996; Williams *et al.*, 2001; Fravolini *et al.*, 2002): that response may have been triggered by decreased stomatal conductance at high VPD. Although a VPD effect on this relationship has not yet been reported, the adjustment of Rubisco/PEPc activities seems to be an important mechanism for plants to counteract the reduced *g*_s_ under long-term low water supply (Saliendra *et al.*, 1996) or high VPD. Maintaining a powerful C_4_ pump (at the expense of increased leakiness) might reduce the quantum yield due to the additional ATP requirement for PEP regeneration (Hatch, 1987; Furbank *et al.*, 1990). However, this mechanism may be beneficial for plants to maintain a high WUE_i_ under high VPD or drought stress.

In this work, high N nutrition increased leakiness in short-term exposures to subambient to ambient [CO_2_], but not to elevated [CO_2_] of 800 μmol mol^−1^. This result is at variance with previous reports of a higher leakiness of plants growing with a low N supply (Meinzer and Zhu, 1998; Fravolini *et al.*, 2002). Other studies also reported non-significant effects of N supply on leakiness (Fravolini *et al.*, 2002; Wang *et al.*, 2012). In the present study, the plants of the low N treatment had a nitrogen nutrition index of ~0.8, which indicated that N was just growth limiting. High N supply led to a NNI of 1.3, and increased plant growth rate via stimulated tiller production (data not shown), but leaf-level net assimilation rates of young leaves were not affected. These results indicate that our low N and high N treatment represented rather ‘nearly-adequate’ and ‘copious supply’ of N for leaf-level photosynthesis. High N supply increased leakiness, in correspondence with a lower *A*_sat_/CE ratio, again indicating the involvement of the relative activities of Rubisco/PEPc. This interpretation was further supported by the positive correlation between N_mass_ and CE, and simultaneous absence of such a relationship between N_mass_ and *A*_sat_. These results suggest that under copious N supply to *C. squarrosa*, the activity of PEPc was more strongly enhanced relative to that of Rubisco, in line with experimental results on *Amaranthus retroflexus* (Sage *et al.*, 1987). Thus, our results are also in agreement with the hypothesis that C_4_ plants have less plasticity in increasing the amount of Rubisco compared with C_3_ plants, due to spatial restrictions in bundle-sheath cells (Sage and McKown, 2006).

Another potential origin of the variance in leakiness is related to the bundle-sheath conductance (*g*_bs_=*ϕV*_P_/(*C*_bs_−*C*_m_)). However, current understanding on *g*_bs_ is limited due to the lack of a methodology for quantification of *g*_bs_ that is independent of gas exchange measurements. Furthermore, it is unknown how VPD may affect the properties of bundle-sheath cell walls, limiting to some extent discussions of potential mechanisms. A recent study demonstrated that *Miscanthus×giganteus* plants grown at high N supply had higher bundle-sheath area per unit leaf area, which might increase *g*_bs_; however, estimates of leakiness did not differ between N levels (Ma *et al.*, 2016). Such results highlight the potential complexity of influences on leakiness by biochemical/physiological and anatomical features. In our study, treatments had little effect on individual leaf area and no effect on thickness (Liu *et al.*, 2016), indicating that effects of leaf anatomy may have been minor. Across all treatments, leakiness measured under growth conditions was negatively related to *A*_sat_/CE, again suggesting that variations of leakiness across treatments was related to the balance between C_3_ and C_4_ cycles.

### Response of bundle-sheath leakiness to short-term variation of CO_2_

The response of *ϕ* to short-term variation of [CO_2_] has been predicted by model analyses (von Caemmerer and Furbank, 1999; Kiirats *et al.*, 2002; Ghannoum, 2009; Yin *et al.*, 2011). Although these models were designed for estimating gas exchange rate rather than leakiness, the present results supported the theoretical predictions. So far, only a small number of studies investigated the response of *ϕ* to dynamic changes of [CO_2_]. A study using the on-line ∆ method showed no response of *ϕ* of *F. bidentis* to dynamic changes of CO_2_ (Pengelly *et al.*, 2012), in agreement with the finding of an earlier study on several species (Henderson *et al.*, 1992). However, in the latter study, each leaf was measured at only two or three levels of CO_2_. Those results suggested a rapid regulation of C_3_ and C_4_ cycle rates in response to changing CO_2_ concentrations. The present results indicate that this kind of regulation did not occur to the same extent during short-term CO_2_ responses in *C. squarrosa*. Clearly, the response of *ϕ* to short-term variation of CO_2_ should be studied in detail with a greater number of species.

### *Implications on the ecophysiology of* C. squarrosa

This work questions the interpretation of leakiness as a simple indicator for efficiency of C_4_ photosynthesis or physiological fitness of C_4_ plants, as increased leakiness was related to the enhancement of WUE_i_ in *C. squarrosa*. The latter observation was consistent across N and VPD treatments (long-term response) and short-term variation of CO_2_ levels. This result was in line with the theoretical prediction of the simplified model of C_4_ discrimination: WUE_i_ is positively correlated to leakiness when ∆>4.4‰, i.e. when *ϕ>*0.37. This relationship indicates a potential trade-off between quantum yield and water use efficiency: high leakiness under VPD stress might decrease the efficiency of energy use, but may be a condition for maintaining the effectiveness of CCM and minimizing photorespiration at a very low *g*_s_ in this species. This mechanism is meaningful for C_4_ species in arid or semi-arid grasslands, where shading is uncommon but high VPD or drought stress is a common attribute of the habitat. Considering the reduction of Rubisco specificity for CO_2_ relative to O_2_ by the increase of leaf temperature, this trade-off may be important for C_4_ plants in the context of global warming. Observational evidence has indeed shown that this C_4_ grass has increased its relative abundance in vast areas of Inner Mongolian grasslands during regional warming in the last decades (Wittmer *et al.*, 2010).

## Supplementary data

Supplementary data are available at *JXB* online.

Fig. S1. Relative humidity and leaf-to-air vapour pressure deficit in the leaf cuvette during the measurement of CO_2_ response curves.

Fig. S2. Minimum mesophyll conductance to CO_2_ (*g*_mmin_=*A*/*C*_i_) and estimated *g*_m_ in response to *C*_i_.

Fig. S3. Photosynthetic light response curves of leaves of *C. squarrosa*.

Fig. S4. *V*_p_ and *V*_c_ in response to *C*_i_.

Fig. S5. Bundle-sheath leakiness in response to *C*_i_, calculated using different models of ^13^C discrimination.

Fig. S6. Correlations between WUE_i_ and bundle-sheath leakiness of *C. squarrosa*.

## Supplementary Material

Supplementary_Figures_S1_S6
