# Supplementary material for: Bundle-sheath leakiness and intrinsic water use efficiency of a perennial C4 grass are increased at high vapour pressure deficit during growth
Source: J Exp Bot. 2016 Nov 18;68(2):321–33. doi: 10.1093/jxb/erw417 (PMC5853292; doi:10.1093/jxb/erw417)

Fig. S1. Relative humidity and leaf-to-air vapour pressure deficit in the leaf cuvette during measurements of $CO_2$ response curves of plants grown at low (V1, open symbols) or high VPD (V2, filled symbols). Dashed lines indicate the relative humidity or VPD of the growth environment of the low VPD treatment (grey lines) and high VPD treatment (black lines). Data are shown as mean ± SE (n = 17-20, data of N treatments were averaged).

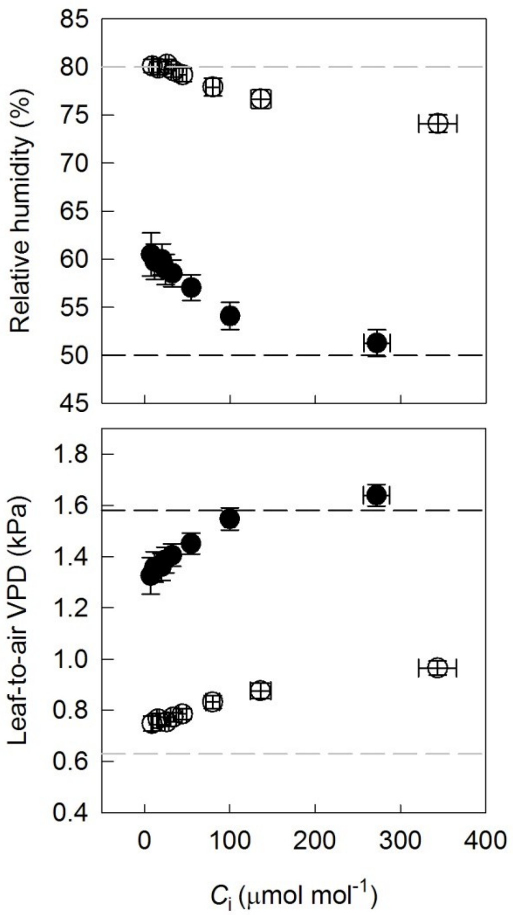

Fig. S2. Minimum mesophyll conductance to $CO_2$ ($g_{m\ min} = A/C_i$) and the estimated $g_m$ in response to short-term variation of $C_i$ under low (N1, circles) or high N supply (N2, triangles) combined with low (V1, open symbols, dushed lines) or high VPD (V2, filled symbols, solid lines). Operating conditions of gas exchange measurements were the same as conditions in growth chambers (leaf temperature 25 °C, PPFD 800 µmol m$^{-2}$ s$^{-1}$, VPD 0.8 kPa for V1 and 1.6 kPa for V2). Data are shown as mean ± SE (n = 5-6).

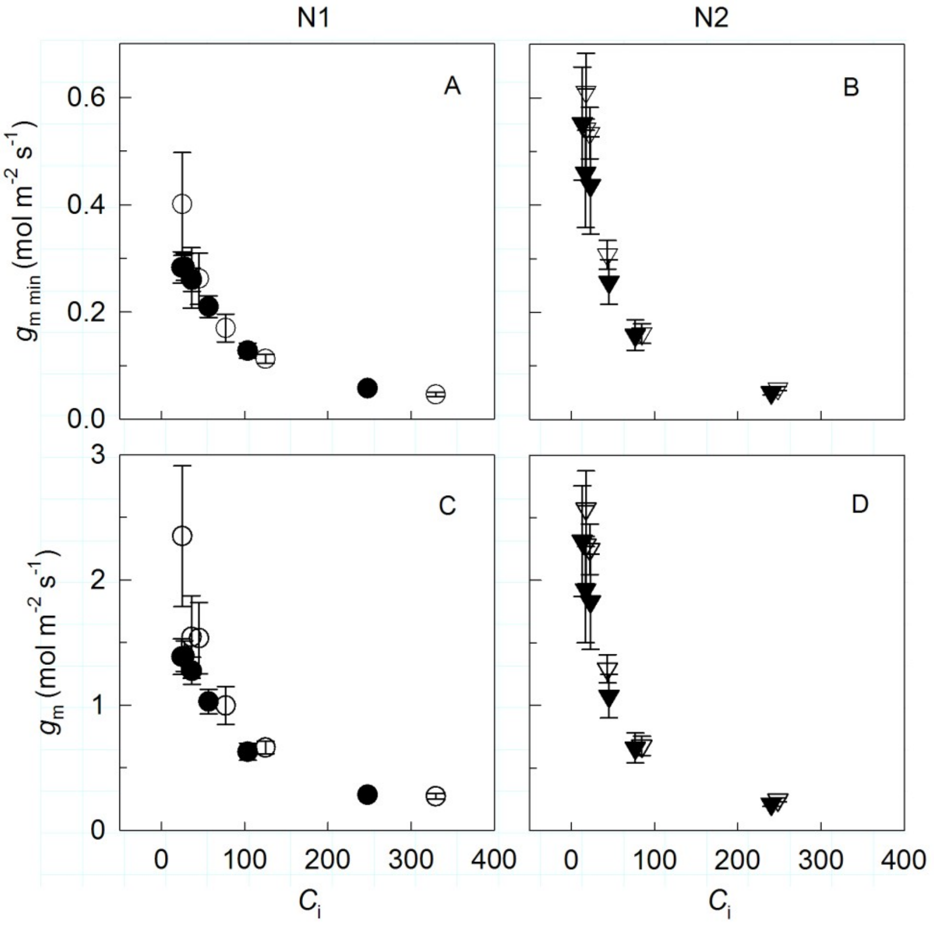

Fig. S3. Photosynthetic light response curves of leaves of *C. squarrosa* grown under low (N1, circles) or high N supply (N2, triangles) combined with low (V1, open symbols) or high VPD (V2, filled symbols). Operating conditions of gas exchange measurements were the same as conditions in growth chambers (leaf temperature 25 °C, [$CO_2$] 390 µmol mol$^{-1}$, VPD 0.8 kPa for V1 and 1.6 kPa for V2). Data are shown as mean ± SE (n = 3-4).

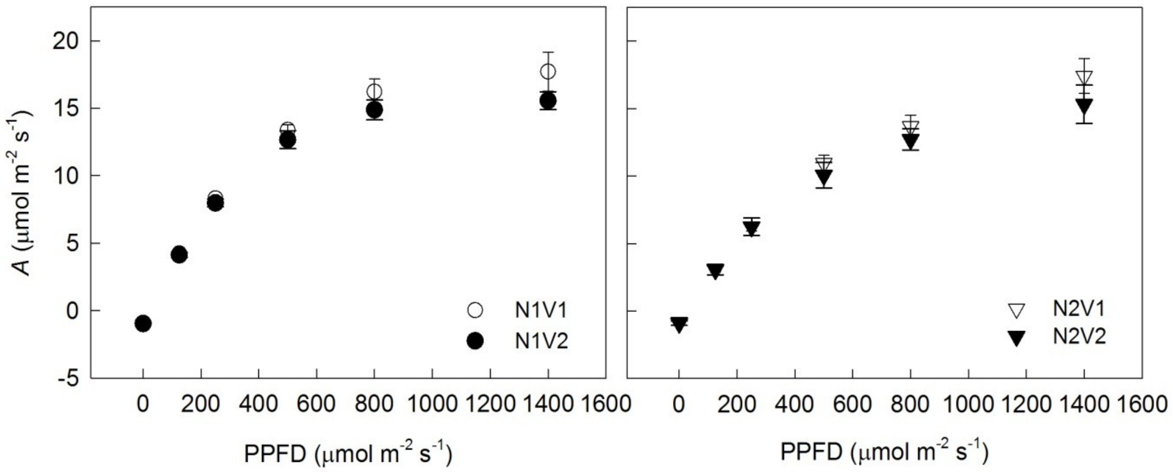

Fig. S4. PEP carboxylation rate ($V_p$, squares) and Rubisco carboxylation rate ($V_c$, triangles) in response to short-term variation of intercellular $CO_2$ ($C_i$) under low (N1, panel A) or high N supply (N2, panel B) combined with low (V1, open symbols, dushed lines) or high VPD (V2, filled symbols, solid lines). $V_p$ and $V_c$ were estimated according to equations in von Caemmerer and Furbank (1999): $V_p = A + 0.5R_L/(1 - \phi)$; $V_c = A + R_L + 0.5V_o = (A + R_L)/(1 - 0.5V_o/V_c)$. For determination of $V_o/V_c$ see 'Materials and Methods'. Operating conditions of gas exchange measurements were the same as conditions in growth chambers (leaf temperature 25 °C, PPFD 800 μmol $m^{-2}$ $s^{-1}$, VPD 0.8 kPa for V1 and 1.6 kPa for V2). Data are shown as the mean ± SE (n = 5-6). The regressions were fitted using a function of $y = y_0 + a$ Ln (x) for $V_p$, and using a function of y = a (1-exp(-bx)) for $V_c$ , all regressions have $r^2 > 0.9$.

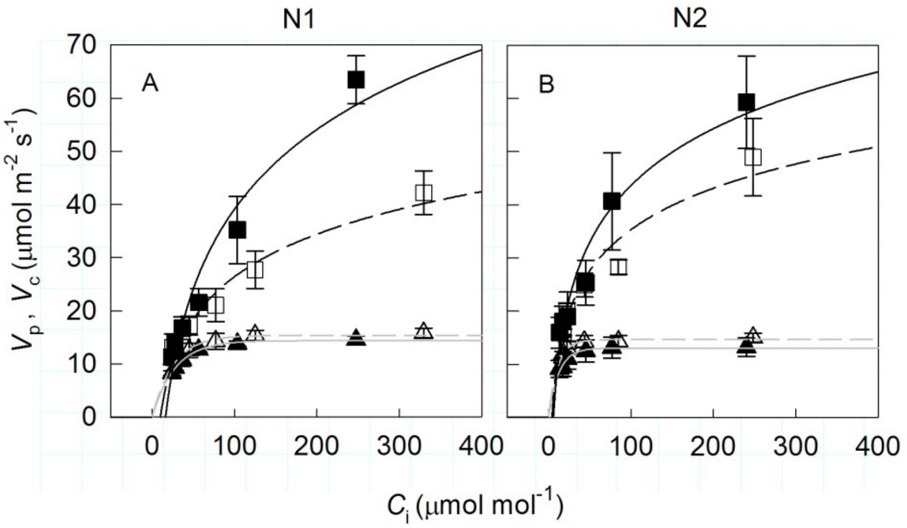

Fig. S5. Dynamic changes of bundle-sheath leakiness in response to short-term variation of $C_i$ under low (N1, circles) or high N supply (N2, triangles) combined with low (V1, open symbols, dushed lines) or high VPD (V2, filled symbols, solid lines). Bundle-sheath leakiness was calculated using different models of $^{13}C$ discrimination: the first model (panel A and B) assumes a constant gm/gmmin ratio across $C_i$ levels, $g_m$ at ambient $CO_2$ is 0.45 mol m$^{-2}$ s$^{-1}$; the 2rd model (panel C and D) assumes infinite $g_m$ across $C_i$ levels; the 3rd model (panel E and F) assumes $g_m$ = infinite, $CO_2$ and $HCO_3^-$ are not at equilibrium, and $V_p/V_h = 0.23$; the 4th model assumes $g_m$ = infinite, $C_{bs}/(C_{bs} - C_m) = 1$, and $C_m/(C_{bs} - C_m) = 0$. Operating conditions of gas exchange measurements were the same as conditions in growth chambers (leaf temperature 25 °C, PPFD 800 µmol m$^{-2}$ s$^{-1}$, VPD 0.8 kPa for V1 and 1.6 kPa for V2). Data are shown as mean ± SE (n = 5-6).

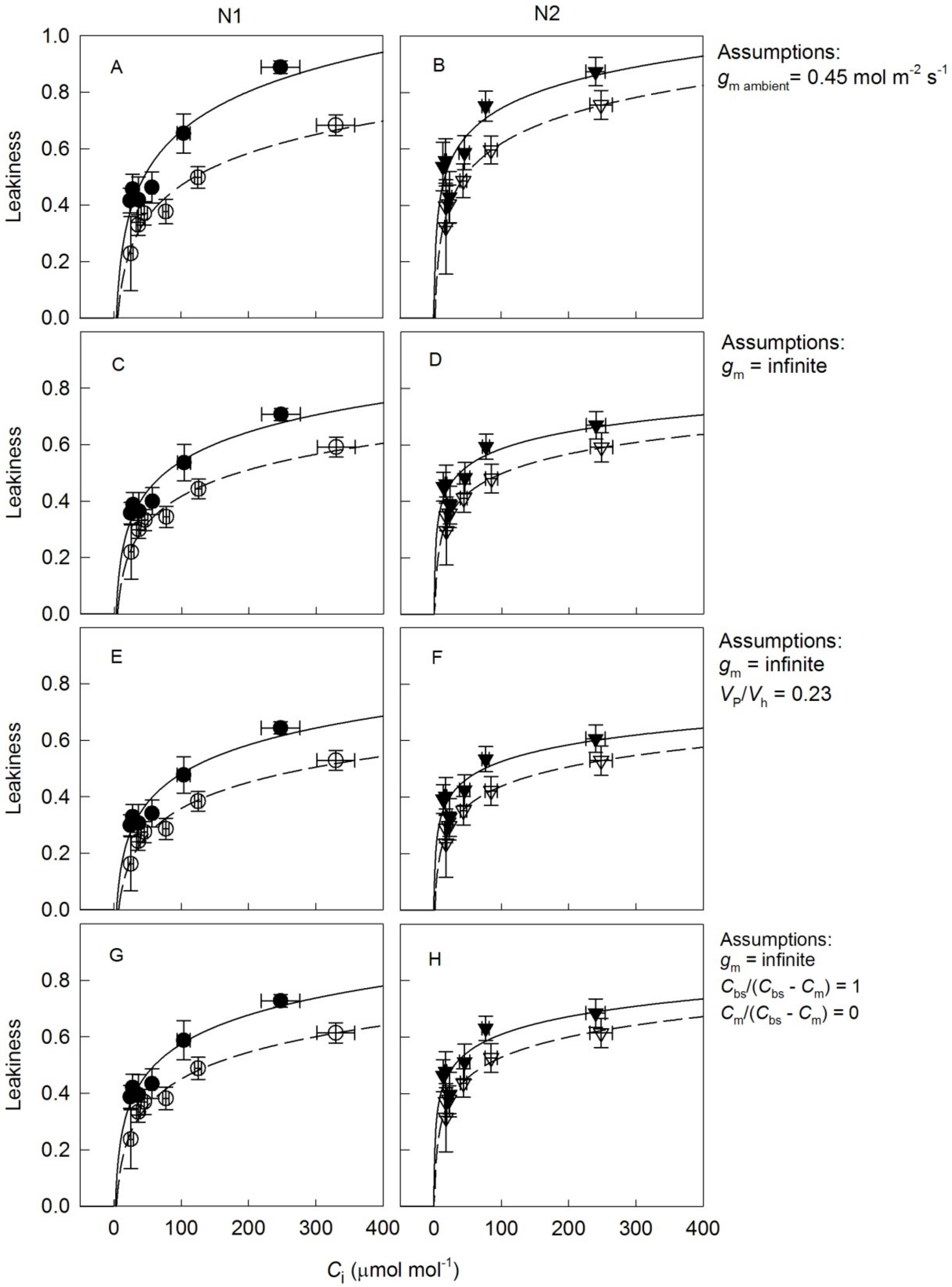

N1
N2
A
B
C
D
E
F
G
H
Leakiness
1.0
0.8
0.6
0.4
0.2
0.0
0
100
200
300
400
$C_i$ (μmol mol$^{-1}$)
Assumptions:
$g_{m\ ambient}$ = 0.45 mol m$^{-2}$ s$^{-1}$
Assumptions:
$g_m$ = infinite
Assumptions:
$g_m$ = infinite
$V_P/V_h$ = 0.23
Assumptions:
$g_m$ = infinite
$C_{bs}/(C_{bs} - C_m)$ = 1
$C_m/(C_{bs} - C_m)$ = 0

Fig. S6. Correlations between $WUE_i$ and bundle-sheath leakiness of *C. squarrosa*. Data were obtained during short-term change of $CO_2$ concentration, each symbol represents mean ± standard error (n = 5-6). Symbols distinguish plants grown under low (N1, circles) or high N supply (N2, triangles) combined with low (V1, open symbols) or high VPD (V2, filled symbols). Operating conditions of gas exchange measurements were the same as conditions in growth chambers (leaf temperature 25 °C, PPFD 800 μmol $m^{-2}$ $s^{-1}$, VPD 0.8 kPa for V1 and 1.6 kPa for V2).

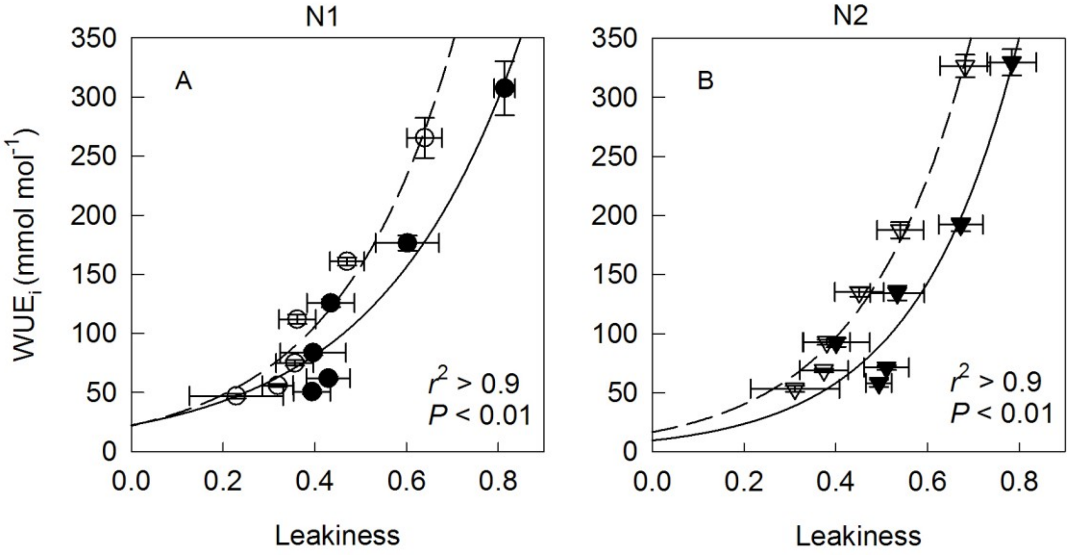

Supplement: Supplementary_Figures_S1_S6 [file erw417_suppl_supplementary_figures_s1_s6.pdf]
